# Establishment of a *Drosophila* AD model

**DOI:** 10.14440/jbm.2016.61

**Published:** 2016-06-07

**Authors:** Xingjun Wang, Yu Zhao, Yujia Hu, Pu Ren, Ying Sun, Xiaowei Guo, Xirui Huang, Yumeng Zhu, Xinhong Chen, Yu Feng, Lei Xue

**Affiliations:** Institute of Intervention Vessel, Shanghai 10^th^ People's Hospital, Shanghai Key Laboratory of Signaling and Diseases Research, School of Life Science and Technology, Tongji University, 1239 Siping Road, Shanghai 200092, China

**Keywords:** *Drosophila*, APP, Alzheimer’s disease, AD, model

## Abstract

Alzheimer’s disease (AD) is the most common form of dementia that affects people’s health greatly. Though amyloid precursor protein (APP) has been implicated in the pathogenesis of AD, the exact role of APP and its underlying mechanism in AD progression have remained largely elusive. *Drosophila melanogaster* has been extensively used as a model organism to study a wide range of human diseases including AD. In this protocol, we expressed full length human APP in the *Drosophila* nervous system and examined its effect on locomotion and choice ability. We found that expression of APP produced locomotion defects in larvae as measured by plate crawling ability assay (PCA), and in adult flies as monitored by plate cycling ability assay (CLA). In addition, expression of APP results in male courtship choice (MCC) defect, since wild-type males court preferentially toward young virgin females over old ones, while APP-expressing males failed to show this preference. This protocol enables us to screen for novel AD candidate genes as well as therapeutic compounds to ameliorate the disease.

## INTRODUCTION

Alzheimer’s disease (AD), first described more than 100 years ago [1-3], is one of the most common type of neurodegenerative diseases in the world, yet little progress has been achieved in finding an effective therapy for the disease. The main characteristics of AD syndrome include locomotion disadvantage and impairment in the cognitive ability [4-6]. The amyloid precursor protein (APP) has been implicated in the development of AD [7-9], yet the underlying mechanism of APP’s pathological role in AD progression remains largely elusive. APP encodes a single-pass transmembrane protein that can be cleaved at different sites to generate a series of fragments including the Aβ peptides, N-APP and APP intracellular domain (AICD). Though the extracellular senile plaques (SP) containing the Aβ peptides have been proposed as a major cause of AD [10-12], the pathological role of Aβ peptides in AD progression has been challenged. Clinical studies found no direct correlation between Aβ plaques load in the brain of AD patients and the severity of clinical symptoms [13], and that clearance of amyloid plaques from AD patients using an immunization protocol against Aβ peptides failed to prevent progressive neurodegeneration [14]. Besides, N-APP and AICD were shown to play pivotal role in regulating neuronal cell death [15-17], and thus, may also contribute to APP-induced neurodegeneration. *Drosophila* has been extensively used as a powerful genetic model system to study a wild range of human diseases including neurodegenerative diseases, and several *Drosophila* AD models have previously been established based on eye-specific or pan-neuronal expression of Aβ peptides [18-21]. Here we report a new *Drosophila* AD model in which the full length human APP is expressed in the nervous system. In this model, APP expressing larvae and flies exhibit a couple of behavioral disabilities, including locomotion and choice defects, which can be used for genetic screen to identify novel genes that modulate the expression, transportation, stability, modification and cleavage of APP, or genes that act as downstream mediators of APP’s pathological functions. In addition, this model can be used to test or screen potential therapeutic molecules for AD.

### Development of the protocol

Recently, we have developed an *in vivo* protocol to study the pathological functions of APP in *Drosophila* [17,22,23]. In the method, we expressed the full length human amyloid precursor protein (APP) in the central nervous system, and observed locomotion defects in both larvae and adult flies, which we termed “Locomotion decline”. Expression of APP also eliminated male flies’ courtship preference for young virgin females over old ones, which we termed “Male courtship choice (MCC) defect”. The APP-induced AD-like symptoms provide an effective *Drosophila* AD model that could be used for genetic screen to identify unknown factors that interact with APP, and for drug screen to find potential therapeutic compounds for AD.

**Figure 1.**
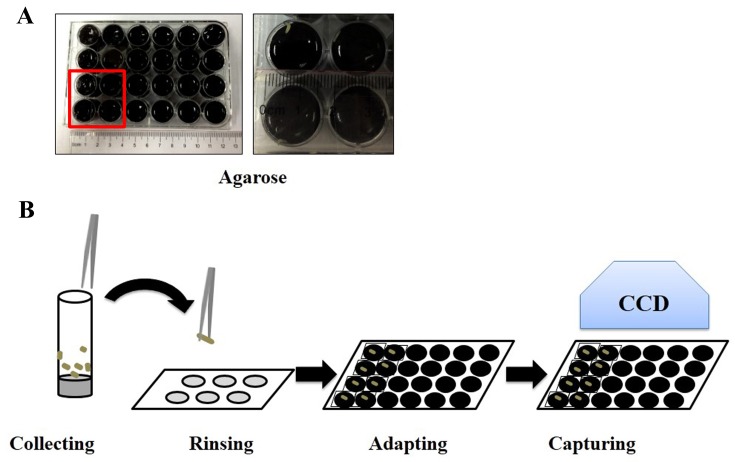
**Locomotion assay for larvae. A.** The light image of a 24-well plate with 3% agarose/black ink is shown. The right panel is the magnification of red box in the left one. **B.** The flowchart of the process for the plate crawling ability assay is shown.

**Figure 2.**
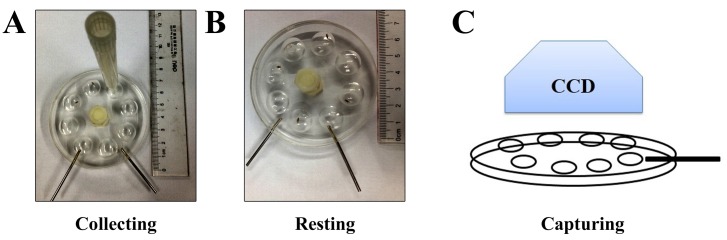
**Locomotion assay for flies. A-C.** The process of cycling ability assay is shown. The plastic tube (A) is used to transfer flies into the cycling chamber (B), and the flies’ locomotion is recorded (C).

Expression of APP in the same set of neurons as *Drosophila* APP homolog APPL by *APPL*-Gal4 [24-27] resulted in neuronal cell death and impaired locomotion ability in the third instar larvae, which were rescued by depletion of the transcriptional factor FoxO [17]. We measured the larval locomotion defects by examining their plate crawling ability (**Fig. 1A** and **1B**), which we termed “PCA” assay. This assay allows us to check the roles of APP in early stages of animal development. As most patients begin to suffer from AD at a late stage of lifetime, an average age of 65 years, known as “Late onset Alzheimer’s Disease” [28], we also examined the locomotion ability of adult flies at different ages. Climbing assay has been commonly used to test the locomotion ability of adult flies, however, in this setup, flies are subjected to a mechanical force by being dropped to the bottom of test vials and subsequently climb up the sides of the vials [29]. This mechanical force may impose certain artificial side effect on flies’ development. To avoid the side effect, we placed adult flies into an 8-well round chamber and recorded their cycling ability, termed “CLA” assay (**Fig. 2**), which monitors flies’ horizontal locomotion ability without any external influence. Expression of APP in the nervous system driven by *APPL*-Gal4 resulted in a strong defect in CLA assay.

To monitor the choice defects of AD flies, we examined males’ courtship choice toward different aged virgin females. We found that wild type males, when given both young and old females, tended to mate with younger ones [22], while expression of APP in the nervous system by *elav*-Gal4, *fruitless*-Gal4 or *Gr33a*-Gal4, impaired males preference to younger females [22,23]. This male courtship choice assay, termed “MCC” assay, was used to confirm that the transcriptional factor FoxO is required for APP-induced choice defects [23].

### Applications of the methods

The methods described above demonstrate that expression of human APP in *Drosophila* results in locomotion decline and choice impairment. The phenotypes can be used for genetic screen to identify novel genes that modulate the activities or functions of APP, and for *in vivo* drug screen to obtain potential therapeutic compounds for AD. The methods may also be adapted for other neurodegenerative diseases.

### Experiments design

The protocol of the experiments can be broken down into several independent parts: larval plate crawling ability (PCA) assay, adult cycling ability (CLA) assay and male courtship choice (MCC) assay.

#### Larva plate crawling ability (PCA) assay

Agarose/black ink (1:1) mix were added to the bottom of wells in a 24-well plate (the diameter is 1.5 cm) to generate a black background and subsequently covered with a layer of 3% agarose. 3rd instar wandering larvae were collected from the vials and transferred into the agarose wells individually. The wells were covered by slides and larvae were allowed to adapt to the agarose surface for 5 min before videotaping. Larvae moved on the agarose surface randomly and their moving tracks were videotaped by a HDR-CX270 digital video camera (Sony). Each larva was videotaped for 8 minutes, repeated for 5 times with an interval of 1 min. Multiple larvae could be videotaped at the same time (we usually test 10 larvae each time). The moving tracks and velocity were calculated by the Noldus EthoVision® XT software (Noldus Information Technology) (**Fig. 1A** and **1B**).

#### Adult cycling ability (CLA) assay

To examine adult cycling ability, we generated a special round plate with 8 chambers (The diameter is 1.5 cm) (**Fig. 2A**). Flies were transferred individually into the chambers where they could walk freely without any artificial interruption (**Fig. 2B**), and their moving tracks were recorded for 6 min (**Fig. 2C**). Noldus EthoVision® XT software (Noldus Information Technology) was used to analyze the videos and produce data such as the total distance, average velocity and instantaneous speed. The *APPL*>APP flies showed a significant difference from the control flies in these aspects. Thus, the assay could be recognized as another *Drosophila* AD screening model.

#### Male courtship choice (MCC) assay

**Figure 3.**
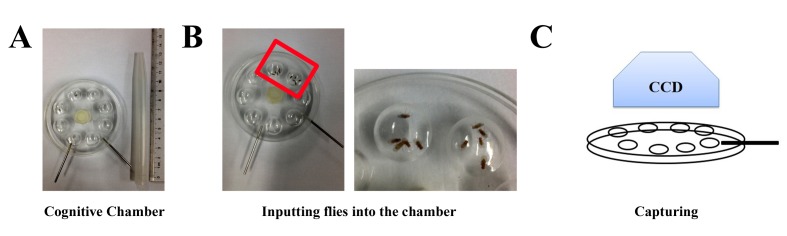
**Cognitive assay for flies. A.** The chamber for cognitive assay is shown. **B.** One naïve male is placed together with 2 young females and 2 old ones into the chamber for assay. The right panel is a magnification of the left one. **C.** The courtship is captured by video.

To test adult choice ability, we used the same chambers as that in the adult cycling assay. In this assay, one naïve male was placed together with two young virgin females (3 days old) and two old ones (30 days old) in the same chamber (**Fig. 3A** and **3B**). We previously found that APP expression induced strong neuronal cell death [17] and abolished male’s mating preference to young females [22]. Thus, this method is a novel assay to test the choice ability of *Drosophila*. Using this method we recently confirmed that the transcriptional factor FoxO mediates APP-induced cognitive impairment [23], which was consistent with the data in the locomotion assay.

### Limitations

While many genes, such as tau [30-34], APOE [35-37] and γ-secretase [38,39], have been associated with AD, the methods described here only aim to investigate the pathological function of APP in the progression of AD. To investigate the role of other genes in AD, additional models should be developed.

## MATERIALS

### REAGENTS

• Transgenic fly strains used in the methods included *APPL*-Gal4 [40], *elav*-Gal4, *fruitless*-Gal4 [22] or *Gr33a*-Gal4 [23], *UAS*-APP [17], *w1118* and *Oregan* R [22].
• Standard fly food (1 L dH_2_O contains 135 g brown sugar, 85 g corn flour, 8 g dried yeast, 7 g agar, 4 ml propionic acid). **CAUTION:** Propionic acid is toxic. When adding the propionic acid into the food at the last step, be sure to wear gloves and do not let the propionic acid hurt the skin or eyes.
• Agarose (Biowest® regular agarose G-10, cat. #111860)
• Black ink (Hero Ink from No. 799 Jiahao Rd. Shanghai, China, cat. # Q/VCRL 01). **CAUTION**: Avoid contact and inhalation of the ink. It may be harmful to the eyes and skins.
• 20 × PBS solution (Sangon, Shanghai, China, cat. # SD8117)

### EQUIPMENT

• Culture dish (American Corning, cat. # 430167)
• 6-well plate (American Corning, cat. # 3516)
• 24-well plate (American Corning, cat. # 3524)
• Cycling chamber (manufactured by Tian Bu Chong Ping plastic products factory in Haimen, Jiangsu, China)
• Tweezers (Switzerland, Dumont. cat. # RS-5015)
• HDR-CX270 digital video camera (Sony)
• Nikon software, NIS-Elements D
• Noldus EthoVision® XT software (Noldus Information Technology)

### REAGENT SETUP

#### 3% agarose/ black ink plate

Prepare a 24-well plate, agarose, black ink. Add 1.5 g agarose to 50 ml dH_2_O in a conical flask, dissolve agarose into water by heating in a microwave, carefully add 50 ml black ink into the conical flask, and mix together. Place the mixture into wells of 24-well plates, add a layer of 3% agarose on top of the mixture. **CAUTION**: When adding the hot mixture into the plate, be sure to wear a glove and do not let the mixture touch your skins or eyes.

**CRITICAL**: The 3% agarose/black ink plate should be freshly prepared before each experiment and the plate should be put into the 25°C incubator before testing to make sure the temperature of the plate is 25°C. Be sure to add a layer of 3% agarose onto the surface of the mixture.

#### 1% PBS buffer

Add 25 ml 20 × PBS Solution into 475 ml dH_2_O to obtain 1 × PBS solution.

## PROCEDURE

### For the larva locomotion assay

1. Outcross the *APPL*-Gal4 line with *w1118* line and *UAS*-APP line respectively.
**CRITICAL STEP**: Outcross the *UAS*-APP line with *APPL*-Gal4 3 days before the cross of *APPL*-Gal4 line with *w1118* line, for the expression of APP driven by *APPL*-Gal4 will delay the egg laying.
**CRITICAL STEP**: Choose *APPL*-Gal4 females to cross with naïve *w1118* line and *UAS*-APP line, for *APPL*-Gal4 is located on the X chromosome, thus you can get more intended larvae.
2. Maintain the crosses at 25°C. Transfer the parent flies to a fresh vial every 2 days.
**CRITICAL STEP**: Transfer the parent flies to a new vial with freshly cooked food for the *APPL*>APP larva grows poorly in the old food.

### Plate crawling assay. ●TIMING 4 h (Fig. 1)

3. Put 24-well plates into 25°C incubator 2 h before the testing.
4. Carefully collect 3^rd^ instar female larvae from the vials.
**CRITICAL STEP**: Be sure to collect female larvae.
5. Quickly rinse the larva in 1 × PBS Solution. ●TIMING 10 s.
**CRITICAL STEP**: Be sure not to leave the larva in the PBS buffer for too long.
6. Carefully place the rinsed larva into a 3% agarose/black ink well, let it adapt to the plate and cover each well with an object slide to restrict the larva in the well. ●TIMING 5 min
7. Videotape the moving track of the larva for 8 min with a HDR-CX270 digital video camera (Sony). ●TIMING 8 min
8. Stop videotaping, take a 1 min break. ●TIMING 1 min
9. Go back to step 7 to repeat the procedure for 4 times. ●TIMING 36 min
10. Exclude the larva after testing.
11. Repeat the test with a new larva of the same genotype.
12. A total of 10 larvae are tested for each genotype. Note: Basically, the same results could be achieved by using 5 larvae.
13. Calculate the moving track and velocity of each larva by Noldus EthoVision® XT software (Noldus Information Technology).

### For the adult locomotion assay

14. Outcross the *APPL*-Gal4 line with *w1118* line and *UAS*-APP line at 25°C.
15. Maintain the flies at 25°C. Transfer the parent flies to a fresh vial every 3 days.
**CRITICAL STEP**: *APPL*>APP flies enclose nearly 16 days after egg laying. So it would be a better option to setup the cross between *APPL*-Gal4 and *UAS*-APP 6 days before that between *APPL*-Gal4 and *w1118*, in order to get the offsprings at the same time.
16. Isolate the virgin flies on the day of eclosion.

### Adult cycling ability assay. ●TIMING ~30 days (Fig. 2)

17. On the 5^th^ day post eclosion, transfer one single fly into a round observation chamber (1.5 cm diameter, 0.3 cm depth) using a plastic tube without CO_2_ anesthesia in order to avoid any side effects on behaviors.
**CRITICAL STEP**: Avoid injury to the flies, be careful when transferring the flies, especially the APP-expressing flies.
18. After 1 min of resting, record the cycling of flies for 6 min with an HDR-CX270 digital video camera (Sony).
19. Stop videotaping and take a 1 min break. Repeat step 18 for 2 times. ●TIMING 14 min
20. Repeat the experiment with another fly of the same genotype.
21. A total of 10 adult flies are tested for every genotype. Note: Basically, the same results could be achieved by using 5 adult flies.
22. Calculate the moving track and velocity of each fly by Noldus EthoVision® XT software (Noldus Information Technology)
**CRITICAL STEP**: Carry out the assay in a 25°C room.

### For the adult choice ability assay

23. Collect both *Oregan* R and *w1118* virgin females and maintain them at 25°C on a 12-h dark/light cycle, transfer the flies to a fresh vial every 3 days. ●TIMING 30 d
**CRITICAL STEP**: Be sure to prepare old females 30 days before the cognitive assay
24. Outcross *elav*-Gal4 or *fruitless*-Gal4 or *Gr33a*-Gal4 line with *w1118* line and *UAS*-APP line respectively, and place the crosses at 25°C on a 12-h dark/light cycle.
25. Collect both *Oregan* R and *w1118* virgin females and maintain them at 25°C on a 12-h dark/light cycle for 3 days before the cognitive assay. ●TIMING 3d
**CRITICAL STEP**: Be sure to prepare young females 3 days before the cognitive assay
26. Collect control and APP-expressing naïve males on a CO_2_ pad and maintain the males at 25°C on a 12-h dark/light cycle for 3 days before the cognitive assay. Ten males are collected for each genotype. Note: Basically, the same results could be achieved by using 10–20 adult flies.
**CRITICAL STEP**: Control the CO_2_ pressure to make sure it is just enough to keep the flies anesthetized. Too much CO_2_ will cause the flies to fold their wings, which may affect the courtship
27. Place one control or APP naïve male together with 2 old and 2 young females into a round observation chambers (1.5 cm in diameter and 0.3 cm in depth).
**CRITICAL STEP**: Choose the *Oregan* R as the old females and *w1118* as the young females. In the parallel experiments select the *w1118* as the old females and *Oregan* R as the young females.
**CRITICAL STEP**: The behavior assays were performed within 1 h at the beginning of the 12-h illumination half of the light/dark cycle.
28. Record 10 min of the courtship with the HDR-CX270 digital video camera (Sony) and the video was analyzed by the Noldus EthoVision XT software (Noldus Information Technology).

**Figure 4.**
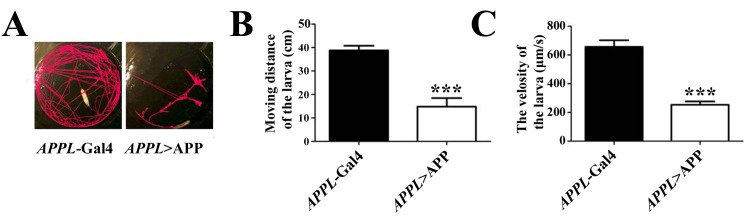
**Expression of APP induces larval locomotion defects. A.** Expression of APP results in restricted moving tracks of larvae. **B** and **C** show the statistic data of the moving distance and velocity of APP-expressing and control larvae in 8 min, respectively. ***, *P* ≤ 0.001. A total of 10 larvae are tested for each genotype. The moving track and velocity of each larva were calculated by Noldus EthoVision® XT software (Noldus Information Technology) and Student’s t-test was used. Genotypes: (A-C) Control groups (*APPL*-Gal4/+), tested groups (*APPL*-Gal4/+; *UAS*-APP/+)

**Figure 5.**
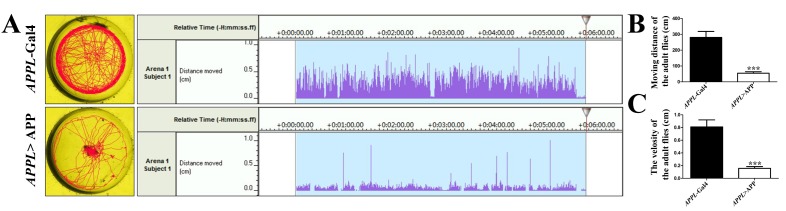
**Expression of APP induces adult locomotion defects. A.** Moving tracks of adult flies are shown. The distance is shown in right panels. The data are analyzed by Noldus EthoVision XT software (Noldus Information Technology). **B** and **C.** show the statistic data of moving distance and velocity, respectively. ***, *P* ≤ 0.001. A total of 10 adult flies are tested for each genotype. The moving track and velocity of each fly were calculated by Noldus EthoVision® XT software (Noldus Information Technology) and Student’s *t*-test was used. Genotypes: (A) Control groups (*APPL*-Gal4/+), tested groups (*APPL*-Gal4/+; *UAS*-APP/+)

**Figure 6.**
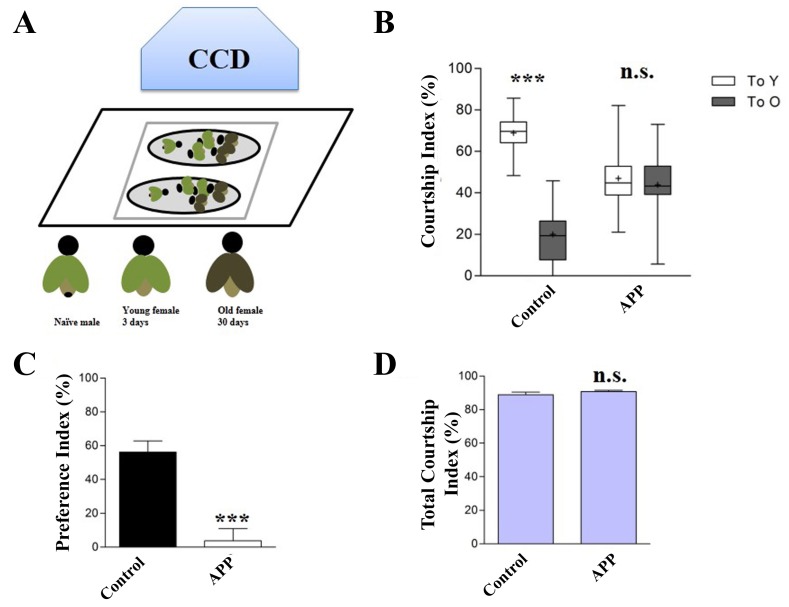
**Expression of APP induced choice defects in adults. A.** A cartoon illustrates the copulation choice assay. A 3-day old naïve male is placed together with 2 young virgin females and 2 old ones in the chamber. **B-D.** Expression of APP abolishes male flies’ preference to younger females (B and C), but not the total courtship ability (D). ***, *P* ≤ 0.001, n.s., not significant. A total of 10 adult male flies are tested for each genotype. Statistical comparisons of the intragroup CIy and CIo in the choice-assays used related-samples Wilcoxon signed-rank test and Mann-Whitney U test was used in the single-pair assays. Kruskal-Wallis test followed by the post hoc Dunn test was used in the preference index (PI) and total courtship index (CIt). Genotypes: Young virgin females (*W1118*, *Oregan* R, respectively); Old virgin females (*Oregan* R, *W1118*, respectively); (B-D) Control groups (*elav*-Gal4/+), tested groups (*elav*-Gal4/+; *UAS*-APP/+).

## ANTICIPATED RESULTS

The locomotion and cognitive assay described in this study not only allow us to study the pathological role of APP in development and behavior, but also provide an effective animal model to screen unknown genes that genetically interact with APP. For example, expression of full length APP in the nervous system (**Fig. S1**) induced larval locomotion defects. When compared with controls, APP-expressing larvae showed more restricted moving track (**Fig. 4A**), much reduced moving distance and velocity (**Fig. 4B** and **4C**). In our previous work we found that these APP-induced larva locomotion defects were suppressed by loss of FoxO [17], indicating this model could be used for genetic study and large scale screen. For the adult locomotor behavior, our established PCA assay provided a novel way to examine adult locomotion ability. We found that similar to the larva plate crawling data, APP-expressing flies (**Fig. S1**) displayed limited moving tracks, reduced distance and velocity compares with the controls (**Fig. 5A-5C**). Importantly, when reared at 17°C to avoid early developmental defect and shift to 25°Cafter eclosion, APP-expressing flies did not show any defect at young age, but displayed locomotion disability in an aging-dependent manner (**Fig. S2**). For the choice assay (**Fig. 6A**), we found that expression of APP in the nervous system abolished males’ copulation preference to younger females (**Fig. 6B** and **6C**), but not the total courtship ability toward females (**Fig. 6D**). This model was used in our recent study to show that FoxO is required for APP- induced choice impairment [23]. We also checked the immediate-recall memory in APP-expressing flies (**Fig. S3**). Though there was no evident morphological defect in the mushroom body of APP expressing flies (**Fig. S4**), expression of APP in the nervous system induced a decline in the memory performance (**Fig. S3**). Collectively, here we have provided a novel *Drosophila* AD model by expressing full length APP and checked the locomotion, choice and memory defects induced by APP, which may provide an economic and effective *in vivo* model for the screening of APP related genes and therapeutic compounds for AD.

## Supplementary information

**Figure S1**. APP protein expression level in larvae and flies.

**Figure S2**. Expression of APP induced aging-dependent locomotion defects.

**Figure S3**. Expression of APP induced immediate-recall memory defects.

**Figure S4**. Expression of APP induced no obvious morphological defects in the mushroom body.
